# Expanding the Functionality of an Autoinduction Device for Repression of Gene Expression in *Bacillus subtilis*

**DOI:** 10.3390/ijms24010084

**Published:** 2022-12-21

**Authors:** Bruna F. Silva, Graciely G. Corrêa, Vitória F. B. Zocca, Flavio P. Picheli, Milca R. C. R. Lins, Danielle B. Pedrolli

**Affiliations:** 1 Department of Bioprocess Engineering and Biotechnology, School of Pharmaceutical Sciences, Universidade Estadual Paulista (UNESP), Rodovia Araraquara-Jau km 1, Araraquara 14800-903, Brazil; 2 Center for Natural and Human Sciences, Campus Santo André, Federal University of ABC (UFABC), Avenida dos Estados 5001, Santo André 09210-580, Brazil

**Keywords:** autonomous control, autoinduction, dynamic regulation, quorum-sensing, riboflavin, *Bacillus subtilis*

## Abstract

Autonomous control of gene expression through engineered quorum-sensing processes is broadly applicable to biosynthetic pathways, including simultaneous control of different genes. It is also a powerful tool for balancing growth and production. We had previously engineered a modular autoinduction device for the control of gene expression in *B. subtilis*. Now, we expand its functionality to repress gene expression autonomously. The engineered R8 promoter responds to AHL accumulation in the culture medium. In a riboflavin-producing strain, the AHL-Lux complex exerts 5-fold repression on the R8-driven expression of the flavokinase/FAD synthetase gene *ribC*, resulting in a higher titer of the vitamin. We engineered a strain able to autonomously induce and repress different genes simultaneously, demonstrating the potential of the device for use in metabolic engineering.

## 1. Introduction

Tools for dynamic control of gene expression are valuable resources to metabolic engineering to optimize the balance between culture growth and biosynthesis of target compounds. The most used strategies for dynamic control are metabolite-sensing regulators and quorum-sensing systems. Both strategies allow for autonomous gene expression control. Metabolite-sensing control is highly specific and pathway-dependent, while quorum-sensing systems are generalist growth-dependent regulatory networks [1,2,3].

**Figure 1:**
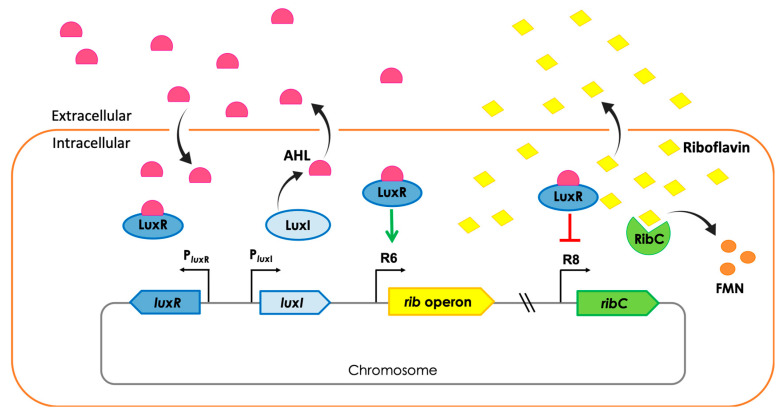
Engineered *B. subtilis* carrying the autoinduction device. In the *B. subtilis*::RFai strain, LuxR binds the LuxI synthesized AHL when the signal reaches the concentration threshold in the culture. The complex AHL-LuxR binds to the *luxbox* in the R6 promoter and activates the transcription of the riboflavin operon (*ribDGEABHT*), generating a quorum-sensing dependent riboflavin overproduction process. Additionally, in the *B. subtilis*::RFai::rRibC strain, AHL-LuxR binds to the *luxbox* in the R8 promoter and blocks the transcription of the flavokinase-encoding *ribC* gene, reducing the cell capacity to convert riboflavin to flavin monophosphate (FMN), leading to a higher accumulation of riboflavin. The *rib* operon and the *ribC* gene are 1.4 Mb distant from each other in the chromosome.

We have previously engineered an autonomous device based on the LuxR/LuxI quorum sensing system to induce gene expression in *Bacillus subtilis*. We used the device to engineer a strain that autonomously induces riboflavin (vitamin B_2_) overproduction [4]. Now, we expand the functionality of the device for the repression of gene expression and applied it to further engineer our riboflavin overproducing strain (Figure 1).

In *B. subtilis*, riboflavin synthesis from GTP and ribulose 6-phosphate is catalyzed by the enzymes encoded by the *ribDGEABHT* genes (*rib* operon). In the cytosol, riboflavin is quickly converted to flavin monophosphate (FMN) which is then converted to flavin adenine dinucleotide (FAD) by the bifunctional enzyme flavokinase (EC 2.7.1.26)/FAD synthetase (EC 2.7.7.2) (RibC or RibFC) [5,6]. Riboflavin accumulates in the cell if its production surpasses the flavokinase capacity to consume it. The excess riboflavin diffuses to the extracellular environment through the cell membrane. We, therefore, targeted the flavokinase gene *ribC* using our autonomous device to repress its transcription and, consequently, improve riboflavin production.

## 2. Results and Discussion

**Figure 2:**
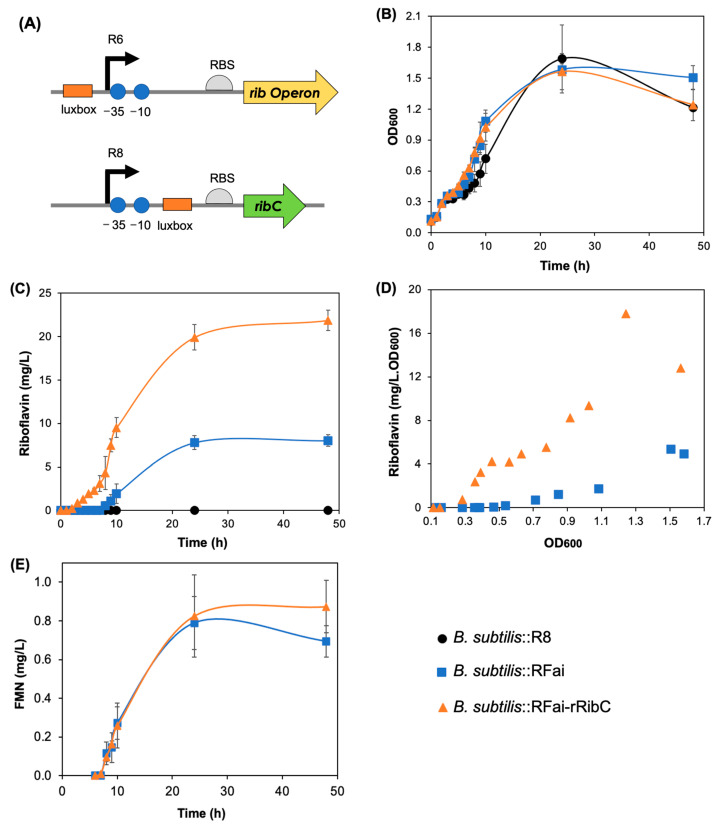
Engineered *B. subtilis* growth and riboflavin production. (**A**) R6 and R8 promoters were engineered to respond to AHL-Lux binding, generating opposite responses. In R6, binding occurs upstream to the −35 region, causing transcription activation. In R8, binding occurs downstream to the −10 region, causing transcription blockage. (**B**) All three engineered strains grew similarly, indicating that neither the autoinduction device nor the blockage of *ribC* expression impairs the population development. (**C**) Riboflavin production by the three engineered strains measured in the culture medium. (**D**) Correlation between the specific riboflavin production and the population density (OD_600_). (**E**) FMN accumulation in the culture medium. Data are presented as mean ± SD (n = 3). Raw data is available in Supplementary Data S1.

*B. subtilis* does not naturally overproduce riboflavin. Laboratory strains such as 168 and KO7 do not produce any detectable flavin in the culture medium. For these strains, flavins can only be detected in concentrated cell extracts. However, it is known that a *B. subtilis* strain producing a mutant flavokinase (RibC G820A) accumulates high amounts of riboflavin to compensate for the reduced enzyme activity. In that strain, the excess of riboflavin can be easily detected in the culture medium [5]. Based on that, we hypothesized that downregulation of the *ribC* gene would have the same effect on the riboflavin biosynthesis. Therefore, we engineered the *ribC* native promoter to be repressed by the AHL-LuxR complex using CRISPR edition. For that, the *luxbox* was inserted downstream of the *ribC* promoter (P*_ribC_*) and upstream of the ribosome binding site of *ribC* (Figure 2A). The engineered promoter was named R8. Two strains were engineered using the R8 promoter: i. *B. subtilis*::RFai, whose *rib* operon is under control of the AHL-LuxR-induced R6 promoter, to generate *B. subtilis*::RFai::rRibC (Figure 1); ii. *B. subtilis* wild-type to generate the control strain *B. subtilis*::R8, which lacks the *luxRI* genes and, therefore, cannot repress the R8 promoter.

To test the improved circuit and validate the AHL-dependent repression of LuxR on the R8 promoter, the two new strains and the *B. subtilis*::RFai parental strain were cultivated for 48 h for growth and riboflavin production measurements. All three strains grew similarly, reaching maximum culture density after 24 h (Figure 2B), demonstrating that R8 engineering is not harmful to the cells. Moreover, the control strain did not overproduce riboflavin, demonstrating that R8 engineering does not affect *ribC* expression in the absence of LuxR and does not disrupt the promoter function. On the other hand, *B. subtilis*::RFai::rRibC is able to overproduce riboflavin to reach 21.8 ± 1.17 mg L^−1^ (58 ± 3.1 µM), an increase of 2.7-fold over the parental strain *B. subtilis*::RFai. Unexpectedly, the LuxR-dependent repression started earlier in the culture course than the LuxR-dependent activation (Figure 2C). It is even possible to identify the two events for the *B. subtilis*::RFai::rRibC strain when the riboflavin specific production is correlated to the OD_600_ (Figure 2D). At OD_600_ = 0.3, riboflavin specific production increases as a first event, possibly due to the *ribC* repression. The specific production stabilizes between 0.4 and 0.6, and then resumes to increase after OD_600_ = 0.7. Around the same point, the riboflavin production by the *B. subtilis*::RFai starts.

The gain in riboflavin production caused by AHL-LuxR repression on *ribC* (14.3 mg L^−1^, 38 µM) closely matches the riboflavin improvement reached through RibC mutation (15.1 mg L^−1^, 40 µM), which decreased the flavokinase activity to about 1% of the wild-type enzyme [5]. In the latter, the riboflavin synthesis increase is assigned to diminished feedback inhibition due to lower FMN levels. FMN is known to trigger the *ribDG* riboswitch, causing transcription termination of the *ribDGEABHT* riboflavin biosynthetic gene cluster [7]. However, abolishing the *ribDG* riboswitch activity does not result in the same level of increase in riboflavin. Deleting the riboswitch terminator sequence resulted in an increase in riboflavin biosynthesis to only 0.8 mg L^−1^ (2 µM), representing a 29-fold increase compared to wild-type [8]. Interestingly, the marked increase in riboflavin did not result in an increase in the FMN levels, neither for the edited riboswitch [8] nor for our AHL-dependent *ribC* repressed strain. After 24 h of growth, we measured 0.78 ± 0.06 and 0.82 ± 0.18 mg L^−1^ of FMN for the *B. subtilis*::RFai and *B. subtilis*::RFai::rRibC, respectively. Indeed, FMN accumulated equally in the medium for both strains over the culture course (Figure 2E). The diminished RibC concentration in the last strain seems to have been proportionally compensated by the increase in the substrate concentration. The K_M_ of RibC for riboflavin was determined as 20.7 ± 0.8 mg L^−1^ (55 ± 2 µM) [9]. Assuming that the riboflavin accumulation in the culture medium results from concentration equilibrium between intra- and extracellular concentrations (passive export), the riboflavin concentration never overcame the enzyme K_M_. According to the Michaelis–Menten kinetics, at substrate concentrations much lower than the K_M_, the reaction rate only depends on the substrate and enzyme concentrations ($V_{0}=\frac{k_{\mathrm{cat}}}{K_{M}}\left[S\right]\left[E\right]$). As the FMN product formation over time did not differ significantly between the strains, the reaction rate in the two strains was equal and the increase in the riboflavin substrate concentration in the *ribC* repressed strain is inversely proportional to the enzyme concentration in the cell. Therefore, we can conclude that, at high cell density (OD_600_ > 1) and low riboflavin concentration ([S] << K_M_), AHL-LuxR exerted 5-fold repression over RibC synthesis in the strain *B. subtilis*::RFai::rRibC compared to the parental strain, since the riboflavin concentration was 5-fold higher at the same time point (at 10 h).

So far, autonomous repression of gene expression has only been built in *B. subtilis* indirectly, using an AHL-LuxR inducible promoter to control the expression of small RNAs designed to repress gene expression in a quorum-sensing-controlled sRNA regulation [10]. We now developed a direct process to autonomously repress gene expression by expanding the functionality of our autoinduction device including a promoter repressed by the AHL-LuxR complex. Our results demonstrate that the quorum-sensing-based autoinduction process can be efficiently used to simultaneously induce and repress different genes in the cell, making for a versatile tool for metabolic engineering in *B. subtilis*.

## 3. Materials and Methods

### 3.1. Bacterial Strains and Growth Conditions

Plasmid cloning and propagation were carried out in *E. coli* Top 10. *B. subtilis* KO7 (BGSC 1A1133) and the derivative strain *B. subtilis*::RFai were used for gene edition and riboflavin production. *B. subtilis*::RFai was previously engineered by inserting the riboflavin operon *ribDGEABHT*, under the control of the S1-R6 autoinduction device, as a single copy into the *amy* locus of the *B. subtilis* KO7 chromosome [4]. The strains were cultivated in Lysogeny Broth (LB) medium enriched with kanamycin and/or mannose when necessary, during transformation and gene editing procedures.

### 3.2. Plasmid Construction and Gene Editing

CRISPR-Cas9 editing plasmid pJOE8999(cas9) was used to edit *B. subtilis* genome [11]. The gRNA sequences were purchased as oligonucleotides forward and reverse strands (Table 1). The complementary strands were annealed by heating an equimolar solution to 98 °C for 10 min and letting it cool down slowly to ambient temperature. Annealed gRNA oligos formed stick ends compatible with *Bsa*I digested plasmid. Plasmid digestion and insert ligation were carried out using Golden Gate Assembly [12]. Donor DNA was PCR amplified from *B. subtilis* genome as 500 bp containing the *truB* (upstream) and *ribC* (downstream) partial gene sequences. The *ribC* forward primer was used to insert the *luxbox* sequence downstream of the P*_ribC_* promoter to generate R8 (Table 1). The two-part donor DNA was assembled using Golden Gate. Finally, the donor DNA was ligated into the plasmid after digestion with *Sfi*I. Correct cloning was confirmed by Sanger sequencing.

The two-step *Bacillus subtilis* transformation procedure was used to transform *B. subtilis* strains. Transformants were selected on LB-agar medium enriched with 5 µg mL^−1^ kanamycin and 0.02% (*m*/*v*) mannose for *cas9* induction. Colonies were then submitted to PCR and DNA Sanger sequencing to identify the correctly edited clones.

### 3.3. Riboflavin Production and Growth Conditions

Engineered *B. subtilis* strains were cultivated in Erlenmeyer flasks (500 mL) filled with 50 mL of medium (40 g L^−1^ sucrose, 1 g L^−1^ yeast extract, 25 g L^−1^ NaNO_3_, 0.333 g L^−1^ KH_2_PO_4_, 1 g L^−1^ Na_2_HPO_4_·12H_2_O, 0.15 g L^−1^ MgSO_4_·7H_2_O, 7.5 mg L^−1^ CaCl_2_, 6 mg L^−1^ MnSO_4_·H_2_O, 6 mg L^−1^ FeSO_4_·7H_2_O, pH 7.0). Cultivations were carried out in triplicate at 37 °C and 220 rpm for 48 h.

### 3.4. Growth Measurement

Cell growth was monitored through optical density at 600 nm. Samples of 200 µL were collected periodically and analyzed in a Tecan Infinite 200 Pro microplate reader (Tecan, Männedorf, Switzerland) using 96-well plates with a transparent bottom.

### 3.5. Flavin Identification and Quantification

Culture samples were centrifuged at 16,000× *g* for 30 min and the supernatant was recovered. Trichloroacetic acid 1% (*v*/*v*) was added to the supernatant and the mixture was centrifugated at 16,000× *g* for 20 min for protein precipitation. The resulting supernatant was filtered through a 0.22 μM PTFE hydrophilic filter and analyzed by high-performance liquid chromatography (LC-20AD Shimadzu, Kyoto, Japan) using a 50 × 3 mm Poroshell 120 EC-C18 column with 2.7 μm particle size (Agilent Technologies, Santa Clara, CA, USA). Flavin peaks were detected at 445 nm (SPD-M20A photodiode detector, Shimadzu, Kyoto, Japan). The mobile phase consisted of 82% (*v*/*v*) of solution A (20 mM formic acid, 20 mM ammonium formate) and 18% (*v*/*v*) methanol. Analysis was carried out at 0.5 mL min^−1^ in isothermal mode at 30 °C [13]. Pure commercial riboflavin and riboflavin 5′-monophosphate (Merck, St. Louis, MO, USA) were used as a standard for calibration.

## Figures and Tables

**Table 1:** DNA sequences used in this study.

ID	Sequence	Use
gRNA(truB 848)_fw	TACGGCTTGGCGATCTATTTCCCT	gRNA forward oligo
gRNA(truB 848)_rv	AAACAGGGAAATAGATCGCCAAGC	gRNA reverse oligo
truB-seq.hom(*Sfi*I)_fw	AAGGCCAACGAGGCCGTATGATAACCATTGAAGACATTGCCC	*truB* PCR forward primer
truB-seq.hom(*Bsa*I)_rv	TCTGGGTCTCTCTATTGTTCGCTTTTTTGCATCAATACTTTTGCCGGCTTTAACAGCCCTTTTTTTGCAGGATGAGGGAAATAGATCGCCAAGCATGTTCCGGATTCAGTAAAGACGGC	*truB* PCR reverse primer
luxbox_ribC(*Bsa*I)_fw	GCTAGGTCTCAATAGACCTGTAGGATCGTACAGGTAAAAAAGGTGACCGTTCTGTGAAGAC	R8 promoter forward primer
luxbox_ribC(*Sfi*I)_rv	AAGGCCTTATTGGCCTGCTCCGTTAATTTTTCTACCATTGTG	R8 promoter reverse primer
R8 promoter sequence	GAGCAGTTTTCTGAAATGACAAGTGGAGACCGTATTGCCGTCTTTACTGAATCCGGAACCTGCTTGGCGATCTATTTCCCTCATCCTGCAAAAAAAGGGCTGTTAAAGCCGGCAAAAGTATTGATGCAAAAAAGCGAACAATAGACCTGTAGGATCGTACAGGTAAAAAAGGTGACCGTTCTGTG	R8 promoter complete sequence

Underlined nucleotides identify *Bsa*I and *Sfi*I recognition sites. In the R8 promotor sequence: gray-shaded sequences identify the −35 and −10 promoter regions and the *luxbox* sequence, respectively. Underlined GTG identifies the *ribC* start codon.

## Data Availability

All data generated in this work is made available in the Supplementary Materials.

## Supplementary Materials

The following supporting information can be downloaded at: https://www.mdpi.com/article/10.3390/ijms24010084/s1, Data S1.
